# Predictors of survival in adults with B-cell acute lymphoblastic leukemia treated with blinatumomab and/or inotuzumab ozogamicin in first salvage

**DOI:** 10.1038/s41375-026-03056-4

**Published:** 2026-07-16

**Authors:** Roberta S. Azevedo, Elias Jabbour, Nitin Jain, Fadi G. Haddad, Partow Kebriaei, Issa Khouri, Elizabeth J. Shpall, Richard Champlin, Omer Karrar, Manuel Maroun, Jayastu Senapati, Eitan Kugler, Rebecca S. Garris, Farhad Ravandi, Hagop Kantarjian, Nicholas J. Short

**Affiliations:** 1 The University of Texas MD Anderson Cancer Center, Department of Leukemia, Houston, USA; 2 The University of Texas MD Anderson Cancer Center, Department of Stem Cell Transplantation and Cellular Therapy, Houston, USA

**Keywords:** Acute lymphocytic leukaemia, Targeted therapies, Haematopoietic stem cells

## Abstract

The role of allogeneic hematopoietic stem cell transplantation (allo-HSCT) in adults with relapsed/refractory (R/R) B-cell acute lymphoblastic leukemia (B-ALL) treated with B-cell–directed immunotherapies remains controversial. We analyzed 172 adults treated in first salvage with blinatumomab and/or inotuzumab ozogamicin (InO)–based regimens to determine prognostic factors and benefit from allo-HSCT after achieving remission. Relapse within 12 months of initial diagnosis (HR 2.21, 95%CI 1.28–4.16) and measurable residual disease (MRD) positivity by multiparametric flow cytometry after cycle 1 of salvage (HR 3.06, 95%CI 1.75–5.33) predicted inferior relapse-free survival (RFS) on multivariate analysis; both factors as well as older age predicted inferior overall survival (OS). Patients who achieved early flow MRD negativity and had late relapse or were primary refractory to frontline therapy did not benefit from allo-HSCT (4-year RFS 60% vs 63% without allo-HSCT, *p* = 0.82), while allo-HSCT improved outcomes in patients with MRD positivity and/or relapse within 12 months (4-year RFS 56% vs. 22% without allo-HSCT, *p* = 0.03). MRD status after cycle 1 of salvage therapy and duration of first remission can risk stratify adults with R/R B-ALL; those with early MRD negativity and late relapse or primary refractory disease may have favorable outcomes without consolidative allo-HSCT.

## Introduction

The historical outcomes of adult patients with relapsed/refractory (R/R) acute lymphoblastic leukemia (ALL) treated with cytotoxic chemotherapy were poor, with a median overall survival (OS) ranging from 3 to 9 months [1–5]. Prognosis was influenced by several factors, including age, number of prior lines of therapy, time to relapse, site of relapse, response to salvage therapy, and performance of allogeneic hematopoietic stem cell transplantation (allo-HSCT) [1–4]. Durable survival in R/R ALL was observed only in patients who achieved a complete remission (CR) with salvage therapy followed by allo-HSCT [2, 6, 7]. In a retrospective analysis of patients with R/R ALL treated with different salvage chemotherapy regimens, no patient without allo-HSCT survived more than one year after relapse compared with a 38% OS rate in those who underwent allo-HSCT after first salvage therapy [4].

However, the development of B-cell–directed immunotherapies, such as blinatumomab and inotuzumab ozogamicin (InO), and the subsequent advent of CD19-targeted chimeric antigen receptor (CAR) T-cell therapy has significantly improved outcomes in adults with R/R B-cell ALL (B-ALL) [8–14]. In this new treatment era, the universal benefit of allo-HSCT has been challenged [15]. Among patients treated with single-agent InO, long-term survival is driven largely by those who proceeded to allo-HSCT, although the risk of post-transplant complications, including sinusoidal obstruction syndrome/venous occlusive disease (SOS/VOD), are not negligible [16–18]. In contrast, *post hoc* analyses of patients treated with blinatumomab for measurable residual disease (MRD) clearance suggest that some patients achieving MRD negativity by multiparameter flow cytometry (MFC) may experience long-term survival without allo-HSCT [19–21]. Similarly, in the R/R setting, some patients who achieved remission with blinatumomab were able to maintain durable responses without undergoing transplantation [9, 22, 23]. Response to CAR T-cell therapy has also been shown to be associated with long-term survival, regardless of whether patients receive consolidation therapy in the form of allo-HSCT [24–26].

In our prior analysis of patients with R/R B-ALL treated with a combination of mini–hyper-CVD (mini-hyperfractionated cyclophosphamide, vincristine, and dexamethasone alternating with mini-methotrexate and cytarabine) and InO, with or without blinatumomab, a landmark analysis found no survival benefit from allo-HSCT among those who achieved remission, with a 3-year OS of 54% in both groups [27]. In this current study, we aim to evaluate the outcomes of patients with R/R B-ALL who achieved remission after receiving blinatumomab and/or InO as part of first salvage therapy, and to identify subgroups of patients who are more or less likely to benefit from consolidation with allo-HSCT.

## Methods

### Study design and participants

This retrospective study evaluated factors predicting outcomes in patients with R/R B-ALL after first salvage therapy. Eligible patients were adults with R/R B-ALL who achieved CR or CR with incomplete hematologic recovery (CRi) following first salvage therapy incorporating blinatumomab and/or InO at our institution. Patient data were collected in accordance with the Institutional Review Board of The University of Texas MD Anderson Cancer Center, and the study was conducted in accordance with the Declaration of Helsinki.

### Treatment and response to therapy

Patients received first salvage therapy with a regimen that included blinatumomab and/or InO, either as single-agent or in combination with chemotherapy. Patients were included whether they received salvage therapy on a clinical trial or as standard of care. All patients with Philadelphia chromosome (Ph)-positive B-ALL also received a tyrosine kinase inhibitor (TKI) as part of their salvage regimen.

CR was defined as bone marrow (BM) blasts < 5%, absence of extra-medullary disease, and recovery of peripheral blood counts (absolute neutrophil count [ANC] ≥ 1 × 10^9^/L and platelet count ≥ 100 × 10^9^/L). CRi was defined as the same as CR, but without complete peripheral blood count recovery (ANC < 1 × 10⁹/L and/or platelet count < 100 × 10⁹/L). MRD assessment was performed on fresh BM aspiration samples using clinically validated MFC, as previously described [28]. MRD negativity was defined as undetectable leukemic blasts by MFC at a sensitivity of 1 x 10^–4^. In more recently treated patients, the clonoSEQ assay for next-generation sequencing (NGS) of IG/TR gene rearrangements was used for MRD measurements at a sensitivity of 1 x 10^–6^, as previously reported [29].

### Statistical analysis

Continuous variables were described as medians and ranges, and categorical variables were reported as frequencies and percentages. Median follow-up was calculated using the reverse Kaplan-Meier method. OS was defined as the time from achieving CR/CRi to death from any cause. Relapse-free survival (RFS) was defined as the time from achieving CR/CRi to either relapse or death for any cause. Patients alive at last follow-up were censured. The Kaplan-Meier method was used to generate survival curves, and comparisons between groups were performed using log-rank test. Cumulative incidence of relapse (CIR) was defined as the time from achieving CR/CRi to relapse, with death in CR/CRi as a competing event. Non-relapse mortality was defined as time from CR/CRi to death without relapse, with relapse considered a competing event. CIR and NRM were estimated using the competing risks method, and Fine and Gray proportional subdistribution hazards model was used for comparison. For analysis evaluating the impact of allo-HSCT on outcomes, a landmark analysis was performed using a prespecified landmark time of 6 months. Cox proportional hazards models were used to perform univariate and multivariate analysis for RFS and OS with allo-HSCT included as a time-dependent covariable, and results were reported as hazard ratio (HR) with 95% confidence interval (CI). All analyses were performed using R statistical software (version 4.4.2).

## Results

### Study population characteristics

From September 2010 to April 2025, 203 patients received first salvage therapy with blinatumomab- and/or InO-based regimens for R/R B-ALL. Among them, 172 patients (85%) achieved CR/CRi and were included in this analysis. Baseline characteristics are summarized in Table 1. The median age at first salvage was 48 years (range, 18–88). Thirty-four patients (20%) had primary refractory disease, defined as failure to achieve a morphologic remission after one or two cycles of cytotoxic chemotherapy, 45 patients (26%) experienced early relapse (within 12 months of initial diagnosis), and 93 (54%) had late relapse ( ≥ 12 months after initial diagnosis). BM involvement was present in 168 patients (98%); 28 (17%) had extramedullary disease (EMD) with BM involvement, and four (2%) had isolated EMD. The median BM blast percentage at relapse was 64% (range, 0–97).

A total of 137 patients (80%) had Ph-negative, and 35 (20%) had Ph-positive B-ALL. Thirty-two patients (19% of the entire cohort; 23% among Ph-negative patients) had high-risk cytogenetics, defined as low-hypodiploid/near-triploid, complex or tetraploid karyotypes, or *KMT2A* rearrangement. Among 100 tested patients, 28 (28%) had Ph-like B-ALL, and 24 of 111 patients (22%) had *TP53* mutation. Overall, 70 of 125 patients (56%) with cytomolecular profiling were classified as having high-risk genomics, defined by the presence of high-risk cytogenetics, Ph-like disease, and/or a *TP53* mutation.

Frontline treatment regimens are listed in Suppl. Table 1. Hyper-CVAD (hyperfractionated cyclophosphamide, vincristine, doxorubicin, and dexamethasone alternating with methotrexate and cytarabine) was the most common frontline therapy in patients with Ph-negative B-ALL (*n* = 103, 75%). Among patients with Ph-positive B-ALL, 26 (74%) received hyper-CVAD in combination with TKI. Fifteen patients (9%) received blinatumomab or InO as part of frontline therapy, and 18 (11%) underwent allo-HSCT in first remission.

### First salvage treatment and response rates

First salvage treatment regimens are shown in Suppl. Table 2. Among Ph-negative patients (*n* = 137), the most common salvage regimen was mini-hyper-CVD (dose-attenuated hyperfractionated cyclophosphamide, vincristine, and dexamethasone alternating with methotrexate and cytarabine) combined with InO (*n* = 61, 45%), followed by mini-hyper-CVD combined with both InO and blinatumomab (*n* = 36, 26%). Nineteen patients (14%) received single-agent InO, and seven (5%) received single-agent blinatumomab. In the Ph-positive cohort (*n* = 35), 16 patients (46%) received blinatumomab combined with a TKI, and 12 patients (34%) were treated with InO plus TKI. Overall, as a first salvage, 65 patients (38%) received both InO and blinatumomab, 71 (41%) received InO without blinatumomab, and 36 (21%) received blinatumomab without InO.

The median number of cycles to achieve best response was one (range, 1–3). One hundred and twenty-five patients (73%) achieved CR, while 47 (27%) achieved CRi.

Overall, 104 patients (60%) achieved MFC MRD negativity after cycle 1 of salvage therapy. Fifty-nine patients (34%) remained MRD-positive, and nine (6%) were not assessed due to isolated EMD or inadequate sample. MFC MRD negativity was achieved in 140 of 166 patients (84%) as best response.

Eighty patients (46%) underwent allo-HSCT following CR/CRi, with a median time from relapse or primary refractoriness to allo-HSCT of 3.0 months (range, 0.4–9.4). Sixteen patients (9%) received CAR T-cell therapy as consolidation while in-ongoing remission, and 76 (44%) did not undergo allo-HSCT or CAR T-cell therapy.

### Allo-HSCT characteristics

Seventy-six of 80 transplanted patients underwent allo-HSCT at our institution and had detailed transplant data available. Transplantation-related characteristics are described in Suppl. Table 3. Thirty-one patients (41%) had a hematopoietic cell transplant-comorbidity index (HCT-CI) score ≥ 3, and 71 (93%) were MFC MRD-negative prior to transplantation. Myeloablative conditioning (MAC) was used in 64 patients (84%), and 18 patients (24% of all transplanted patients and 20% of those who received MAC) received total body irradiation (TBI). Post-transplant cyclophosphamide (PTCy)-based graft-versus-host disease (GVHD) prophylaxis was used in 27 patients (36%).

### Outcomes and predictors of survival

With a median follow-up of 55 months (95% CI, 48–78), the 4-year CIR, RFS, and OS in the entire cohort were 42% (95% CI, 35–50), 38% (95% CI, 31–46), and 45% (95% CI, 38–54), respectively (Suppl. Fig. 1).

**Fig. 1:**
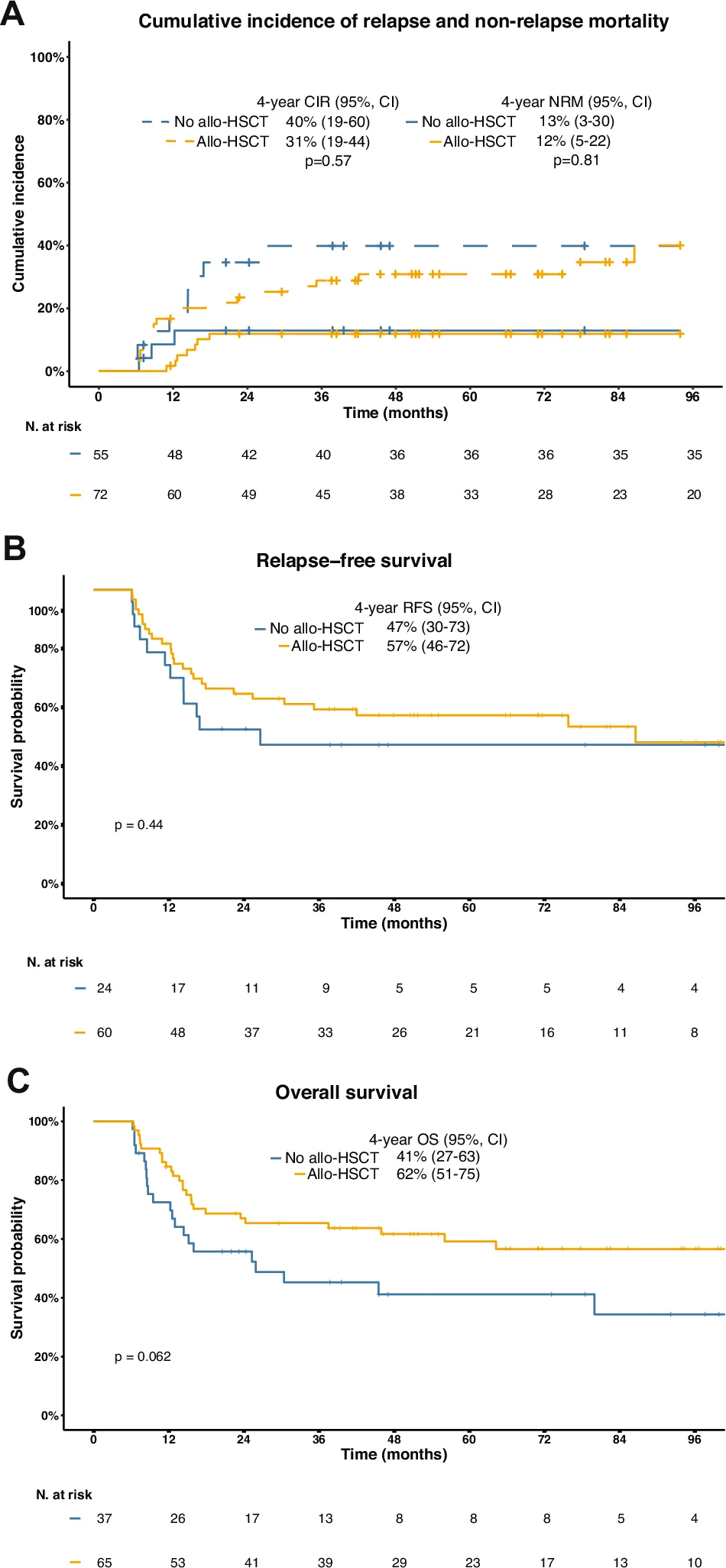
Outcomes stratified by allo-HSCT. Patients who underwent allo-HSCT in first remission or received CAR T-cell therapy were excluded from this analysis. Landmark analysis restricted to patients aged ≤ 70 years. **A** Cumulative incidence of relapse; (**B**) Relapse-free survival; (**C**) Overall survival. Allo-HSCT allogeneic hematopoietic stem cell transplantation.

To assess the impact of allo-HSCT, patients who underwent transplantation in first remission and those who received CAR T-cell therapy as a consolidation were excluded. Patient characteristics were well balanced between those who proceeded to allo-HSCT and those who did not at the time of the landmark analysis (Suppl. Table 4). In a landmark analysis restricted to patients aged ≤ 70 years, those who underwent allo-HSCT had a 4-year CIR of 31% (95% CI, 19–44) compared with 40% (95% CI, 19–60) in non-transplanted patients (*p* = 0.57) (Fig. 1A). The 4-year RFS and OS were 57% (95% CI, 46–72) and 62% (95% CI, 51–75) in transplanted patients, respectively, compared with a 4-year RFS of 47% (95% CI, 30–73) and a 4-year OS of 41% (95% CI, 27–63) in those who did not undergo allo-HSCT (*p* = 0.44 for RFS and *p* = 0.06 for OS) (Figs. 1B and 1C).

**Fig. 2:**
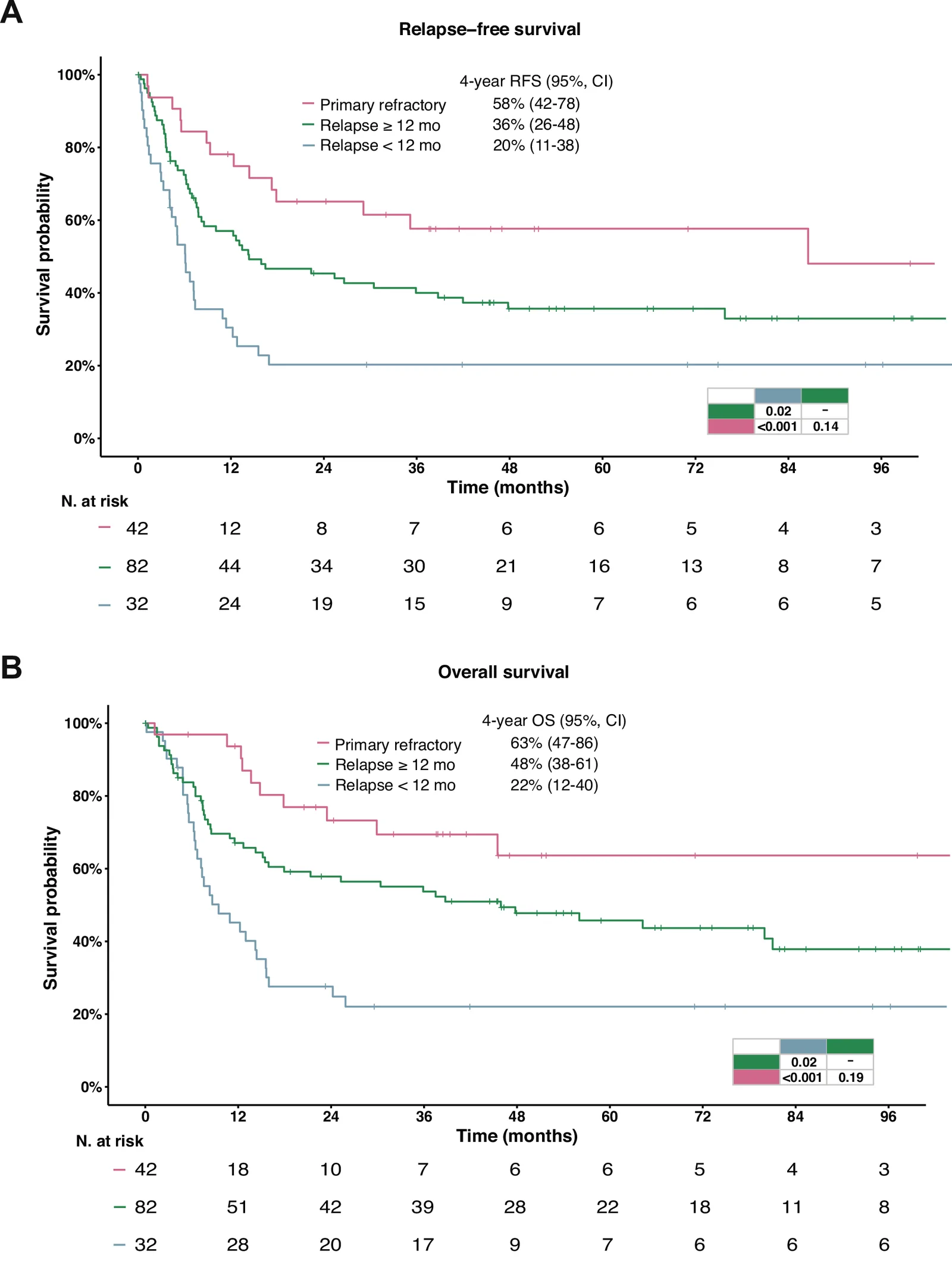
Outcomes stratified by time from diagnosis to relapse. **A** Relapse-free survival and (**B**) Overall survival.

We subsequently performed Cox regression analysis to identify additional factors that may predict survival in the entire cohort. Patients with primary refractory disease had outcomes not statistically different from those with late relapse and were therefore combined for further analyses (adjusted *p* = 0.14 for RFS and 0.19 for OS) (Fig. 2). In the univariate analysis (Suppl. Table 5), the following variables were associated with worse RFS: older age (HR: 1.01, 95% CI, 1.00–1.03, *p* = 0.02), higher white blood cell (WBC) count at relapse (HR 1.01, 95% CI, 1.00-1.02, *p* = 0.04), higher BM blast percentage (HR 1.01, 95% CI, 1.00–1.02, *p* = 0.02), early relapse (versus late relapse or primary refractory disease) (HR: 2.12, 95% CI, 1.39–3.26, *p* < 0.001), and MFC MRD positivity after cycle 1 of salvage therapy (HR: 1.88, 95% CI, 1.24–2.84, *p* = 0.003). For OS, older age (HR: 1.02, 95% CI, 1.01–1.03, *p* = 0.002), early relapse (HR: 2.31, 95% CI, 1.48–3.60, *p* < 0.001), and MFC MRD positivity after cycle 1 of salvage therapy (HR: 1.71, 95% CI, 1.10–2.65, *p* = 0.02), were associated with worse outcomes, while allo-HSCT as a time-dependent variable (HR: 0.63, 95% CI, 0.40–0.99, *p* = 0.046) was associated with superior OS. Ph-negative B-ALL with high-risk genomics was not associated with inferior RFS or OS compared with the standard-risk cytomolecular group. In a subanalysis of different cytomolecular groups, only patients with *KMT2A* rearrangement had worse outcomes compared with those with standard-risk disease (Suppl. Fig. 2). Similar findings were observed in analyses restricted to patients with Ph-negative B-ALL (Suppl. Table 6). However, in patients with Ph-positive B-ALL, the association of time to relapse and MFC MRD positivity after cycle 1 of salvage therapy with outcomes could not be demonstrated, possibly due to the limited sample size (Suppl. Table 7).

In the multivariate analysis, only early relapse (HR: 2.21, 95% CI, 1.23–3.96, *p* = 0.007) and MFC MRD positivity after cycle 1 of salvage therapy (HR: 3.06, 95% CI, 1.75–5.33, *p* < 0.001) were independently associated with inferior RFS. For OS, early relapse (HR: 2.31, 95% CI, 1.28–4.16, *p* = 0.005), MFC MRD positivity (HR: 2.21, 95% CI, 1.28–3.81, *p* = 0.004), and older age (HR: 1.02, 95% CI, 1.00–1.04, *p* = 0.02) were associated with worse outcomes. Allo-HSCT was not independently associated with either RFS or OS (Table 2).

### Combined prognosis of MRD response and time to relapse and the impact of allo-HSCT

Given the significant independent prognostic impact of MFC MRD status after cycle 1 of salvage therapy and time to relapse, patients were stratified into four groups: MRD-negative and late relapse/primary refractory disease (*n* = 63); MRD-negative and early relapse (*n* = 29); MRD-positive and late relapse/primary refractory disease (*n* = 46); and MRD-positive and early relapse (*n* = 10).

**Fig. 3:**
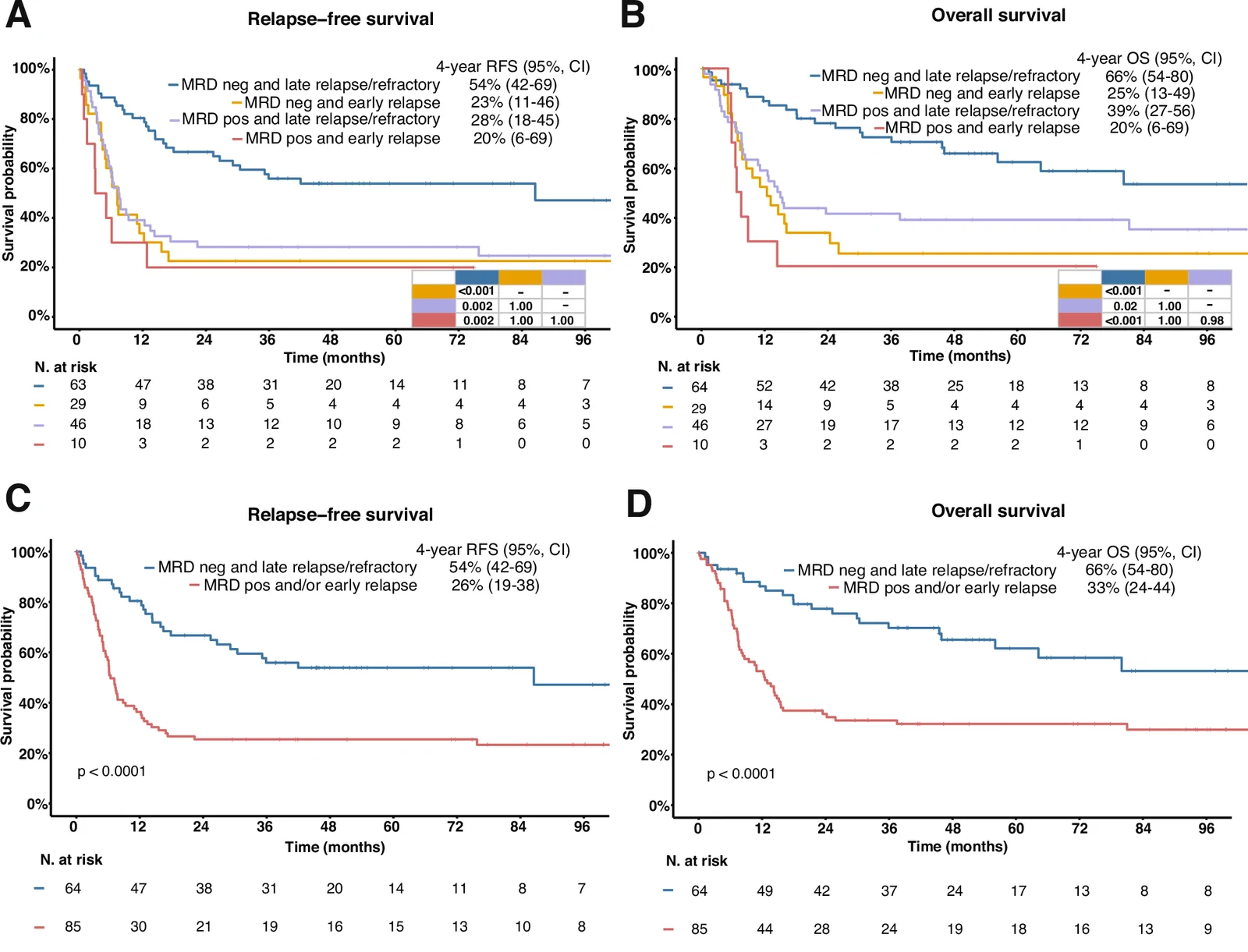
Outcomes in the entire cohort stratified by MFC MRD status after cycle 1 of salvage therapy and time to relapse. **A**, **B** Patients were stratified into four groups: MRD-negative and late relapse/primary refractory disease; MRD-negative and early relapse; MRD-positive and late relapse/primary refractory disease; and MRD-positive and early relapse. **A** Relapse-free survival. **B** Overall survival. **C**,** D** Patients were stratified into two groups: MRD-negative and late relapse/primary refractory disease (“lower-risk” group) and MRD-positive and/or early relapse (“higher-risk” group). **C** Relapse-free survival. **D** Overall survival. MFC multiparameter flow cytometry, MRD measurable residual disease, neg negative, pos positive.

Patients with MRD negativity after cycle 1 and either late relapse or primary refractory disease had relatively favorable outcomes, with a 4-year RFS of 54% (95% CI, 42–69) and a 4-year OS of 66% (95% CI, 54–80), and were subsequently classified as “lower-risk” (Fig. 3A, B). In contrast, all other patients had similarly poor outcomes, with a 4-year RFS of 26% (95% CI, 19–38) and 4-year OS of 33% (95% CI, 24–44) (Fig. 3C, D). Patients with MRD positivity and/or early relapse were therefore classified as “higher-risk”. The results remained largely unchanged after excluding patients with primary refractory disease (Suppl. Fig. 3).

**Fig. 4:**
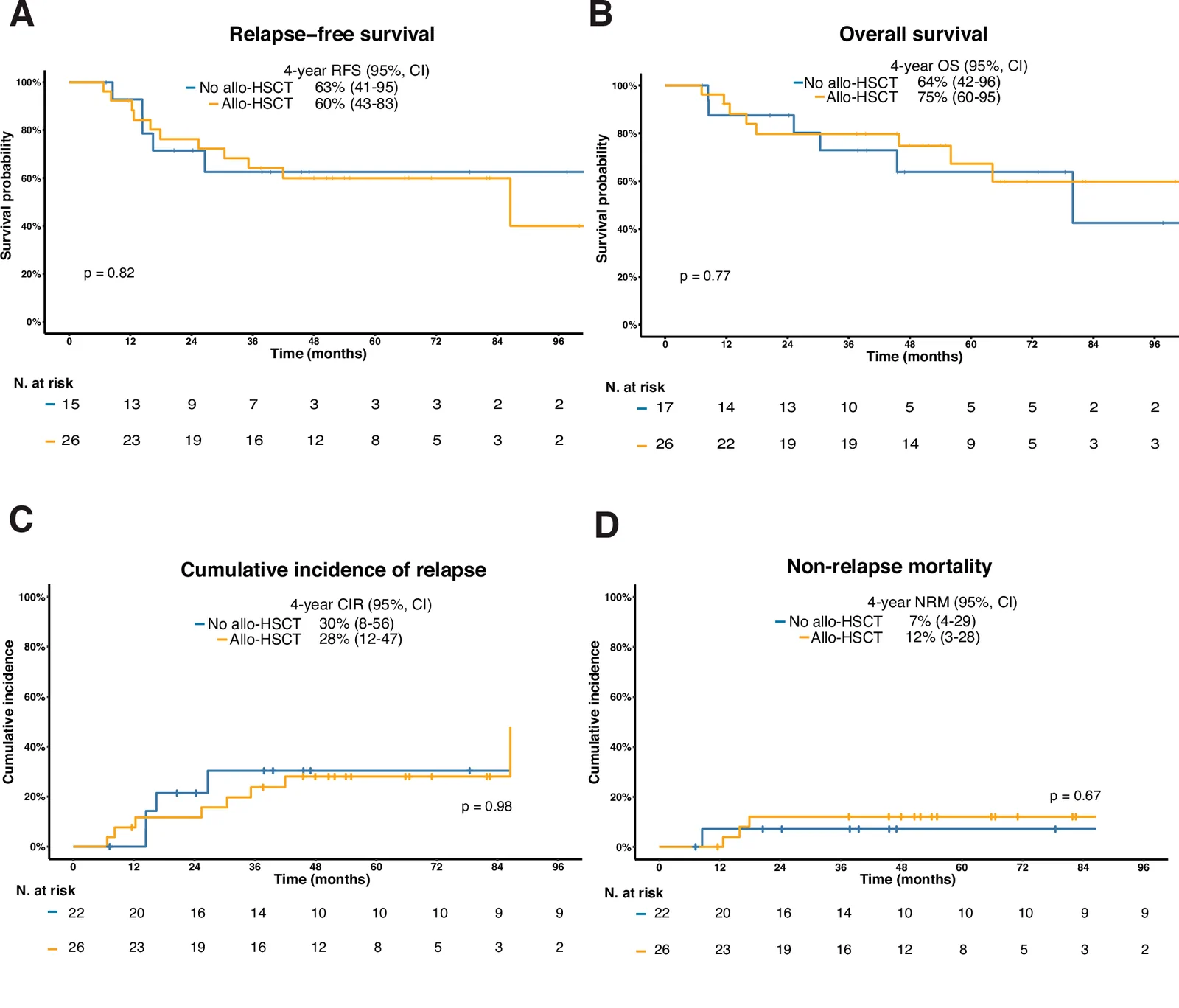
Outcomes in patients with “lower-risk” (defined as MFC MRD negativity after cycle 1 of salvage and either late relapse or primary refractory disease). Patients who underwent allo-HSCT in first remission or received CAR T-cell therapy were excluded from this analysis. Landmark analysis restricted to patients aged ≤ 70 years. **A** Relapse-free survival; (**B**) Overall survival; (**C**) Cumulative incidence of relapse; (**D**) non-relapse mortality. Allo-HSCT allogeneic stem cell transplantation, MFC multiparameter flow cytometry, MRD measurable residual disease.

To evaluate the impact of allo-HSCT in these two risk categories, analyses were restricted to patients aged ≤70 years who would generally be considered eligible for transplantation. Among “lower-risk” patients, allo-HSCT was not associated with a significant improvement in outcomes. In a landmark analysis, the 4-year RFS was 60% (95% CI, 43–83) among patients who underwent allo-HSCT and 63% (95% CI, 41–95) among those who did not (*p* = 0.82) (Fig. 4A). The corresponding 4-year OS rates were 75% (95% CI, 60–95) and 64% (95% CI, 42–96), respectively (*p* = 0.77) (Fig. 4B). The 4-year CIR was 28% (95% CI, 12–47) in transplanted patients and 30% (95% CI, 8–56) in non-transplanted patients (*p* = 0.98), and the 4-year NRM was 12% (95% CI, 3–28) and 7% (95% CI, 4–29), respectively (*p* = 0.67) (Fig. 4C, D).

**Fig. 5:**
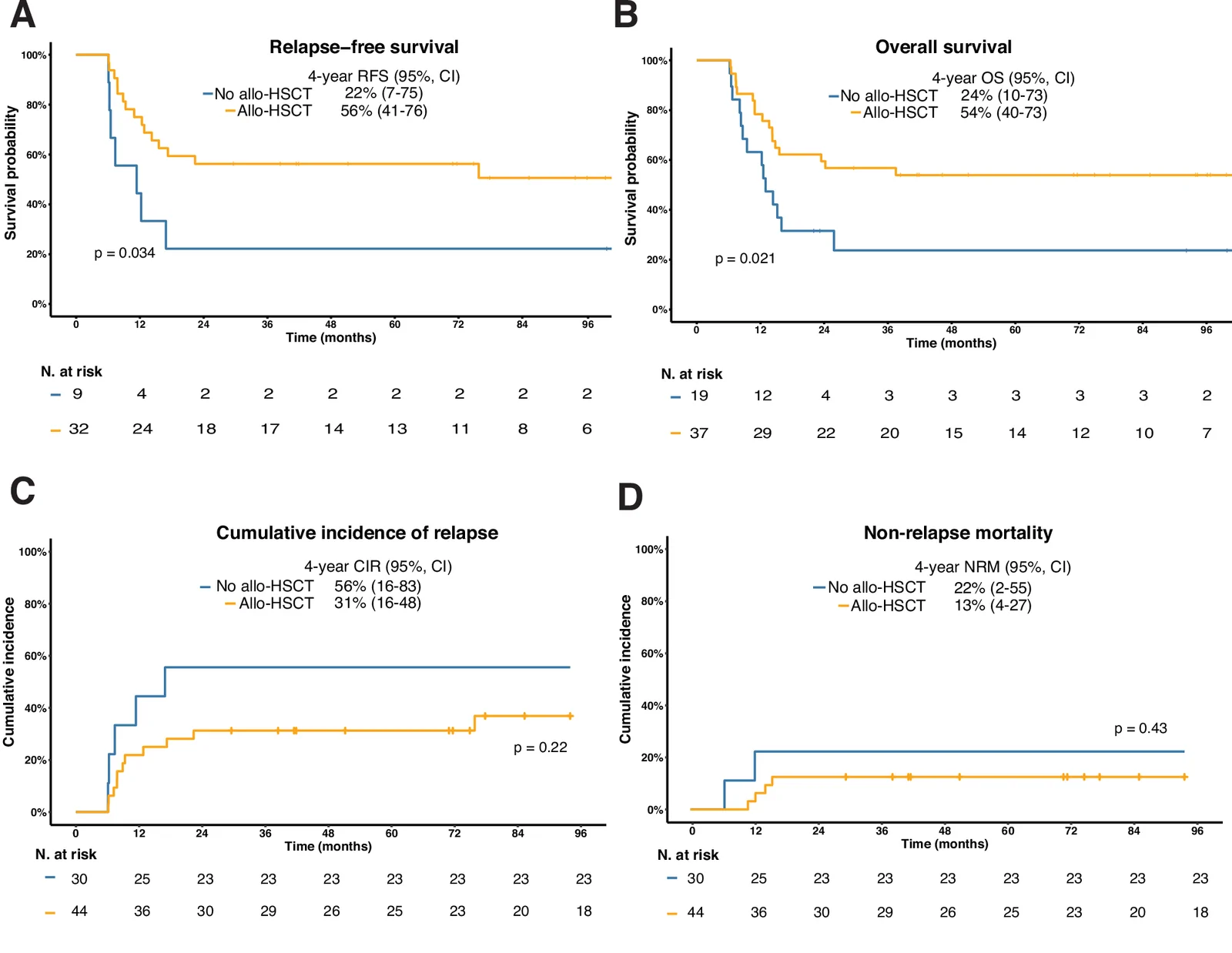
Outcomes in patients with “higher-risk” (defined as MFC MRD positivity after cycle 1 of salvage and/or early relapse). Patients who underwent allo-HSCT in first remission or received CAR T-cell therapy were excluded from this analysis. Landmark analysis restricted to patients aged ≤ 70 years. **A** Relapse-free survival; (**B**) Overall survival; (**C**) Cumulative incidence of relapse; (**D**) non-relapse mortality. Allo-HSCT allogeneic stem cell transplantation, MFC multiparameter flow cytometry, MRD measurable residual disease.

In contrast, allo-HSCT significantly improved outcomes in “higher-risk” patients. The 4-year RFS was 56% (95% CI, 41–76) among patients who underwent allo-HSCT compared with 22% (95% CI, 7–75) among those who did not receive a transplant (*p* = 0.03) (Fig. 5A). OS was similarly improved with allo-HSCT in the higher-risk patients, with 4-year rates of 54% (95% CI, 40–73) in the allo-HSCT group and 24% (95% CI, 10–73) in the non-transplanted group (*p* = 0.02) (Fig. 5B). The 4-year CIR was numerically lower among transplanted patients (31% [95% CI, 16–48] vs 56% [95% CI, 16–83]; *p* = 0.22) (Fig. 5C). The 4-year NRM was 13% (95% CI, 4–27) in transplanted patients and 22% (95% CI, 2–55) among non-transplanted patients (*p* = 0.43) (Fig. 5D). Of note, many patients were ineligible for allo-HSCT because of poor clinical status, and most deaths in remission in the non-transplanted group were attributed to infectious complications.

In patients with Ph-negative B-ALL treated with mini-hyper-CVD and InO, with or without blinatumomab (*n* = 97), subgroup analysis showed outcomes consistent with the overall cohort. Among “lower-risk” patients, the 4-year OS was 77% (95% CI, 54–100) for those who did not undergo allo-HSCT compared with 69% (95% CI, 48–99) for transplanted patients (*p* = 0.82). Among “higher-risk” patients, however, the 4-year OS was 30% (95% CI, 12–77) without allo-HSCT, while transplanted patients had a 4-year OS of 57% (95% CI, 40–83) (*p* = 0.11) (Suppl. Fig. 4).

### Outcomes for patients who received CAR T-cell as a consolidation strategy

Sixteen patients received CAR T-cell therapy after achieving CR/CRi following first salvage, including brexucabtagene autoleucel in 12 patients, and obecabtagene autoleucel and investigational CAR T-cell in 2 patients each. Baseline characteristics are summarized in Suppl. Table 8. The median age at relapse was 43 years (range, 19–76). Primary refractory disease occurred in two patients (13%), early relapse in 3 (19%), and late relapse in 11 (69%). Thirteen patients (81%) received mini-hyper-CVD combined with InO with or without blinatumomab-based treatment. Eleven patients (69%) achieved MFC MRD negativity after cycle 1 of salvage therapy, 3 (18%) were MRD positive, and 2 (13%) were not assessed for MRD. Based on MRD response after first salvage and time to relapse, nine patients (56%) were classified as “lower-risk”, and six (38%) as “higher-risk”. All available patients except one (*n* = 14) achieved MFC MRD negativity before lymphodepletion.

With a median follow-up of 13 months from CAR T-cell infusion (95% CI, 4–not reached [NR]), the 1-year CIR, RFS, and OS were 26% (95% CI, 5–54), 67% (95% CI, 45–100), and 74% (95% CI, 29–100), respectively (Suppl. Fig. 4). Of the three relapses, two occurred in “higher-risk” patients (both at 6 months post CAR T-cell infusion).

### Outcomes based on NGS MRD response after the first cycle of salvage therapy

Nine of 30 patients (30%) achieved NGS MRD negativity after cycle 1 of salvage therapy, all of whom were also MFC MRD negative. Among patients who achieved NGS MRD negativity, eight (89%) had late relapse or primary refractory disease. Two patients (22%) had Ph-positive B-ALL, and among those with Ph-negative B-ALL, two (29%) harbored a *TP53* mutation. Two patients (22%) proceeded to allo-HSCT after achieving remission, and 3 (33%) received CAR T-cell therapy. Notably, only one patient relapsed 7 months after achieving remission with salvage therapy; this patient had early relapse and had previously undergone allo-HSCT during first remission. All other patients who achieved NGS MRD negativity remained alive in ongoing remission at a median follow-up of 16 months. Patient outcomes stratified by NGS MRD status are shown in Suppl. Fig. 6.

## Discussion

In this study, we report the outcomes of a cohort of patients with R/R B-ALL who achieved remission after “optimal” first salvage regimens, including blinatumomab and/or InO-based therapies, and evaluated predictors of relapse and survival, including the role of subsequent allo-HSCT. Overall, outcomes for these patients have improved with modern salvage therapies, with 46% patients alive at 4 years compared with a 3-year OS of 11% in patients with R/R Ph-negative B-ALL treated with cytotoxic chemotherapy [30]. We identified a subgroup of patients who had relatively favorable outcomes and may derive limited benefit from allo-HSCT in first salvage. This “lower-risk” group included patients who achieved MFC MRD negativity after cycle 1 of salvage therapy and either had a relapse ≥ 12 months after initial diagnosis or primary refractory disease. These “lower-risk” patients (comprising 45% of the study population) had encouraging survival, with a 4-year RFS of 60%, and no benefit of allo-HSCT. In contrast, among patients with MFC MRD positivity after cycle 1 of salvage therapy and/or early relapse (i.e., “higher-risk” patients), allo-HSCT was associated with improved outcomes, with a 4-year RFS of 56% compared with 22% among those without allo-HSCT.

Allo-HSCT has been considered the standard approach to achieve durable remission in patients with R/R ALL who respond to salvage therapy [2, 4, 7, 30, 31]. With the advent of monoclonal B-cell-directed antibodies such as InO and blinatumomab, these therapies have become preferred salvage strategies for R/R B-ALL, often as a bridge to transplantation [8, 9]. However, the role of allo-HSCT following remission with these agents remains unclear. In the TOWER study, treatment with single-agent blinatumomab improved OS, despite a similar allo-HSCT rate of 24% in both the blinatumomab and standard chemotherapy arms [9]. Subanalyses of patients who achieved CR/CRi with both intravenous and subcutaneous blinatumomab showed no additional survival benefit from allo-HSCT [22, 23]. In contrast, in patients treated with single-agent InO, a subanalysis of the INO-VATE study demonstrated that those who achieved remission and proceeded to transplantation had superior outcomes, with a 2-year OS of approximately 39%, compared with 13% in patients who achieved CR/CRi but did not undergo allo-HSCT [32]. In our cohort, allo-HSCT was associated with improved survival in univariate analysis. However, on multivariate analysis, allo-HSCT was not independently associated with RFS or OS. These findings should be interpreted with caution given the retrospective design and potential selection bias. In addition, transplant practices continue to evolve, with outcomes following allo-HSCT improving over time, particularly with the adoption of PTCy for GVHD prophylaxis, which was not widely used in our cohort [33, 34].

MRD assessed by MFC is a well-established prognostic marker in both frontline and R/R ALL [35–38]. Our findings are consistent with prior analysis from our group showing that, among patients with R/R B-ALL, achievement of MFC MRD negativity after first salvage therapy is associated with prolonged event-free survival (EFS) and OS [39]. In patients treated with InO, MRD negativity improved progression-free survival (PFS), irrespective of the number of previous lines of therapy [40]. Similarly, among patients receiving blinatumomab, those who achieved MRD negativity were more likely to have long-term survival [37, 41]. While deep NGS-based MRD has prognostic value after frontline therapy and after CAR T-cell therapy in patients with R/R B-ALL, its prognostic significance in patients treated with blinatumomab and/or InO in the salvage setting has not been comprehensively evaluated [29, 42]. In our exploratory subanalysis of patients with available NGS MRD data, 30% achieved NGS MRD negativity after cycle 1 of salvage therapy, and only one relapse was observed; notably, only two of these patients subsequently underwent allo-HSCT. Although limited by small numbers, these findings suggest that achieving early deep molecular remission may be associated with favorable outcomes even in the setting of R/R disease.

Time to relapse is a strong predictor of survival across several cohorts of patients with R/R ALL treated with salvage chemotherapy. A short duration of first remission, defined as relapse within 12–24 months of the initial diagnosis, is associated with a worse prognosis [1–3, 43]. A large multicenter retrospective analysis of patients with Ph-negative B-ALL demonstrated that, among patients without prior allo-HSCT, median OS increased with each additional 6 months of first remission [30]. Interestingly, that study also showed that patients with primary refractory disease had a median OS of approximately 8 months, which was significantly longer than the median OS of 4 months and 5 months observed in those with relapsed disease with and without prior allo-HSCT, respectively [30]. These findings align with our data, indicating that early relapse remains prognostic even in patients treated with B-cell-directed immunotherapies in first salvage. Notably, patients with primary refractory disease –defined as those without a morphological response after one or two cycles but who subsequently achieved remission with B-cell-directed immunotherapies– had relatively better outcomes. The reason for this observation remains unclear, although differences in underlying disease biology may explain this finding.

We showed that the combination of MFC MRD status after cycle 1 of salvage therapy and time to relapse plays a more important role in predicting RFS and OS in R/R B-ALL than either factor alone. Consideration of these two variables may therefore help guide decisions regarding which patients are most likely to benefit from allo-HSCT after achieving remission with B-cell-directed therapy in first salvage. A similar risk-adapted approach is already well established in pediatric B-ALL at first relapse, where early MRD clearance and time to relapse are key determinants of post-relapse strategies [44, 45]. In the prospective UK ALLR3 trial, children with late relapse ( > 6 months after the end of frontline therapy) who achieved MRD < 10^−^^4^ had favorable outcomes with chemotherapy alone and were not routinely bridged to allo-HSCT, whereas those with end of induction MRD ≥ 10^−4^ had significantly inferior survival and were stratified to more intensive consolidation, including transplantation [44, 45].

CD19-directed CAR T-cell therapy has changed the treatment paradigm for R/R B-ALL, leading to deep remission and prolonged survival in responding patients [10, 11, 25]. The use of CAR T-cell as a consolidation strategy may help sustain remission while avoiding some of the toxicity associated with allo-HSCT [46]. Previous studies have consistently demonstrated that a low disease burden prior to CAR T-cell infusion is associated with more durable remissions and lower CAR T-cell-related toxicity in R/R B-ALL [11, 12, 47]. Although longer follow-up is needed, recent data have shown that CAR T-cell consolidation in first CR is associated with encouraging survival, CAR T-cell expansion, and low rates of severe toxicity [48–50]. Among the 16 patients in our cohort who received CAR T-cell as consolidation after achieving remission in first salvage, the 1-year OS was 74%, with only 3 relapses observed. Despite these promising findings, validation in larger cohorts with extended follow-up is required.

We acknowledge that our study has several limitations due to its retrospective design from a single-center institution. We included only patients who achieved CR/CRi after first salvage therapy; therefore, whether these results can be extrapolated to patients treated in later lines is uncertain. Although there was no statistical difference between primary refractory disease and late relapse, we cannot exclude the possibility that predictors of outcomes and the role of allo-HSCT may differ between these populations. In addition, these findings should not be generalized to patients with Ph-positive B-ALL, as the sample size was insufficient for a robust comparison. Separate studies in a larger population of patients with R/R disease are needed to clarify the prognostic factors and role of allo-HSCT in these patients. As this was a retrospective study, selection for allo-HSCT was based on the treating physician’s judgment and was not randomized. Lastly, because our cohort included patients treated over an extended period, only 9% received blinatumomab and/or InO as part of their frontline therapy; thus, the applicability of these findings to patients who relapse after frontline B-cell–directed immunotherapies remains unclear.

In conclusion, our data show that patients with R/R B-cell ALL treated with blinatumomab and/or InO, most of whom also received low-intensity chemotherapy, had a 4-year RFS rate of 38% in the overall cohort. MRD status assessed by MFC after cycle 1 of salvage therapy along with time to relapse allowed stratification of patients into two risk groups and identification of patients who may be more likely to benefit from allo-HSCT. Allo-HSCT was not associated with improved outcomes among patients who were MFC MRD-negative after cycle 1 of salvage therapy and had either a relapse ≥ 12 months or primary refractory disease. In contrast, patients who were MFC MRD-positive after cycle 1 of salvage therapy and/or relapsed within 12 months of the initial diagnosis appeared more likely to achieve long-term survival when consolidation therapy with allo-HSCT was performed. Future studies are needed to assess the role of deep NGS-based MRD assessment and CAR T-cell therapy as consolidation in patients with R/R B-ALL in first salvage, which may further help rationally guide decision-making about consolidative allo-HSCT for these patients.

## Supplementary information

Supplementary Material

## Data Availability

The data that support the findings of this study are available from the corresponding author, N.J.S., upon reasonable request.
